# PCV13 vaccination impact: A multicenter study of pneumonia in 10 pediatric hospitals in Argentina

**DOI:** 10.1371/journal.pone.0199989

**Published:** 2018-07-18

**Authors:** Angela Gentile, Julia Bakir, Verónica Firpo, Enrique V. Casanueva, Gabriela Ensinck, Santiago Lopez Papucci, María F. Lución, Hector Abate, Aldo Cancellara, Fabiana Molina, Andrea Gajo Gane, Alfredo M. Caruso, Alejandro Santillán Iturres, Sofía Fossati, Working Group

**Affiliations:** 1 Epidemiology, Ricardo Gutiérrez Children’s Hospital, Buenos Aires, Argentina; 2 Infectology, Niño Jesus Children’s Hospital, Tucumán, Argentina; 3 Infectology, San Justo Children’s Hospital, San Justo, Buenos Aires, Argentina; 4 Infectology, Victor Vilela Children’s Hospital, Rosario, Santa Fe, Argentina; 5 Infectology, Humberto Notti Pediatric Hospital, Mendoza, Argentina; 6 Infectology, Pedro Elizalde Children's Hospital, Buenos Aires, Argentina; 7 Clinic, Orlando Alassia Children's Hospital, Santa Fe, Argentina; 8 Infectology, Juan Paul II Pediatric Hospital, Corrientes, Argentina; 9 Infectology, Hector Quintana Children's Hospital, Jujuy, Argentina; 10 Infectology, Eva Peron Children's Hospital, Catamarca, Argentina; 11 INEI- ANLIS "Dr. Carlos G. Malbran ", Buenos Aires, Argentina; Public Health England, UNITED KINGDOM

## Abstract

**Introduction:**

In 2012, PCV13 was introduced into the National Immunization Program in Argentina, 2+1 schedule for children <2 years. Coverage rates for 1st and 3rd doses were 69% and 41.0% in 2012, 98% and 86% in 2013; 99% and 89% in 2014, respectively. The aims of this study were to evaluate impact of PCV13 on Consolidated Pneumonia (CP) and Pneumococcal Pneumonia (PP) burden, and to describe epidemiological-clinical pattern of PP during the three-year period following vaccine introduction.

**Methods:**

Hospital-based study at 10 pediatric surveillance units in Argentina. CP and PP discharge rates per 10,000 hospital discharges were compared between the pre-vaccination period 2007–2011 (preVp), the year of intervention (2012) and the post-vaccination period 2013–2014 (postVp).

**Results:**

Significant reduction in CP and PP discharge rates was observed in patients <5 years [% reduction (95%CI)]: 10.2% (6.3; 14.0) in 2012 and 24.8% (21.3; 28.2) in postVp for CP discharge rate; 59.5% (48.0; 68.5) in 2012 and 68.8% (58.3; 76.6) in postVp for PP discharge rate. Significant changes were also observed in children ≥5 years, mainly in PP discharge rate. A total of 297 PP cases were studied; 59.3% male; 31.3% <2 years; 42.9% had received PCV13 in 2012 and 84.5% in posVp. Case fatality rate was 3.4%. PCV13 serotypes decreased from 83.0% (39/47) in 2012 to 64.2% (52/81) in postVp, p = 0.039.

**Conclusions:**

After PCV13 introduction, significant reduction in CP and PP discharge rates was observed in hospitalized children <5 years. In patients ≥5 years, PP discharge rate also decreased significantly.

## Introduction

According to the WHO, 19% of the 10.6 million deaths occurring each year worldwide in children younger than 5 years of age are attributable to pneumonia [1]. *Streptococcus pneumoniae* (Sp) is the main cause of community acquired bacterial pneumonia representing 30–50% of cases [2]. However, quantifying the burden of pneumococcal pneumonia is difficult since Sp is not commonly isolated from blood cultures or pleural fluid, although currently available PCR techniques have improved detection [3]. The prevalence of Sp nasopharyngeal carriage varies from 27% to 85% in developed and developing countries respectively and severe pneumonia accounts for 18% of all cases with high mortality rates in this age group [4, 5].

In recent decades, many population-based and hospital-based epidemiological surveillance studies were carried out in Latin America and the rest of the world to estimate the burden of pneumonia in children based on radiological [Consolidated Pneumonia (CP)] and/or microbiological [Pneumococcal Pneumonia (PP)] definitions [6–9].

Prevalence of Sp serotypes and bacterial resistance were assessed in order to estimate potential benefits of the new pneumococcal conjugate vaccines (PCVs). At the same time, several clinical trials were carried out showing the efficacy and safety of these vaccines against CP and PP in children [10–14].

Based on this evidence and on a local cost-effectiveness analysis, the Argentine Ministry of Health introduced the 13-valent pneumococcal conjugate vaccine (PCV13) into the National Immunization Program in January 2012 using a 2, 4 and 12 months schedule and a two-dose catch-up for all children under 2 years of age born between 2010 and 2011 [15]. Before this date, PCVs had not been included in the National Program. Immunization coverage rates for the 1^st^ and 3^rd^ vaccine doses were 69% and 41% in 2012, 98% and 86% in 2013, 99% and 89% in 2014 and, 92% and 82% in 2015 respectively [16].

The aims of this study were to evaluate the impact of PCV13 in reducing the burden of CP and PP, and to describe epidemiological-clinical patterns of PP observed during the three years following vaccine introduction.

## Methods

### Study design and population

This was an analytical, prospective, active surveillance and retrospective study of pneumonia hospitalizations in children conducted at 10 sentinel pediatric hospitals in Argentina: the Ricardo Gutierrez, Pedro de Elizalde and San Justo Children’s Hospitals in Buenos Aires, Niño Jesus Children´s Hospital in Tucumán, Victor Vilela Children’s Hospital in Rosario, Humberto Notti Pediatric Hospital in Mendoza, Orlando Alassia Children’s Hospital in Santa Fe, Juan Pablo II Pediatric Hospital in Corrientes, Héctor Quintana Children’s Hospital in Jujuy and Eva Perón Children’s Hospital in Catamarca.

All sentinel sites had qualified healthcare staff the infrastructure to diagnose (imaging procedures in line with WHO criteria and microbiological analyses) and treat children hospitalized for pneumonia, as well as the logistics for carrying out surveillance activities.

For the prospective study (2012–2014), study investigators were trained to collect data in a standardized manner using: surveillance records, laboratory and sample submission records, and a case report form containing medical history, nursing reports, and laboratory and microbiology data. Hospital and laboratory databases were used for the retrospective study (2007–2011).

### Inclusion criteria

All children ≤15 years of age with CP hospitalized in one of the sentinel hospitals between January 1^st^ 2007 and December 31^th^ 2014 were included in hospital discharge rate calculations.

Hospitalized children with PP during the three years following vaccine introduction were included in the epidemiological-clinical analysis.

### Data collection

A case report form was completed for each patient with PP containing the following information: participating center, patient’s identification code, admission date, demographic data, age, sex, PCV13 vaccination history, underlying illnesses (such as chronic respiratory diseases, cardiovascular diseases, kidney disorders, metabolic disorders, endocrine disorders -including diabetes-, neurological and neurodevelopmental conditions, genetic disorders, blood disorders, and immunosuppression–including oncohematologic disease, immunosuppression therapy, HIV-), nutritional condition, recent acute respiratory infection (common cold, sore throat, flu-like symptoms, otitis or bronchiolitis in 4 weeks before hospitalization), hospitalizations in the last year, previous antibiotic therapy in the last 3 months, bacteriologic cultures and antibiotic susceptibility, isolation sites, clinical evolution, complications, antibiotic therapy and condition at discharge.

### Definitions

Consolidated Pneumonia was defined as any case with chest X-ray showing dense, white image, cotton wool-like appearance (alveolar infiltrate), involving one or more pulmonary segments, lobes or the whole lung, frequently presenting air bronchogram and sometimes associated with pleural effusion [17].

Pneumococcal Pneumonia was defined as a case of pneumonia in which Sp was isolated from blood or pleural fluid.

### Microbiological studies

Blood and pleural fluid samples were submitted to microbiological testing. Pleural fluid samples were available only for patients who underwent therapeutic thoracocentesis.

*S*. *pneumoniae* isolates were sent to the Clinical Bacteriology Service at the National Institute for Infectious Diseases, National Administration of Health Laboratories and Institutes (INEI-ANLIS) “Dr. Carlos G. Malbran”, for confirmation and capsular serotyping by Quellung reaction. Antimicrobial susceptibility testing was performed by agar diffusion method, and Minimum Inhibitory Concentrations (MIC) were determined using agar microdilution method or *E-test®*, according to Clinical Laboratory Standards Institute (CLSI) standards [18].

### Statistical study

Categorical variables were described as percentages and analyzed through Chi-Squared test with Yates’ correction or Fisher exact test if expected values were <5.

We estimated the number of CP and PP hospitalizations per 10,000 hospital discharges for children ≤ 15 years of age, hospitalized for any cause, per year. Changes in CP and PP rates between the pre-vaccination period 2007–2011 (preVp), the year when PCV13 was introduced (intervention year, 2012) and the post-vaccination period 2013–2014 (postVp) were assessed using the Preventive Fraction in exposed (PFe) formula PFe% = [(Rate preVp–Rate postVp)/ Rate preVp] x 100, with 95% confidence intervals (CIs).

Statistical analysis was performed using Epi Info TM 7 (CDC, Atlanta) and the Open Epi (Open Source Epidemiologic Statistics for Public Health, Version. www.OpenEpi.com, updated on: 2013/04/06). Two-tailed *P* values <0.05 were considered statistically significant.

### Ethics

Data were stored in a restricted database, and personal information was recorded under alphanumeric coding, to keep investigators blind to patients’ identification.

The study was approved by the Research Ethics Committee at each participating hospital.

## Results

### Impact of PCV13 vaccination on burden of CP and PP

#### Discharge rates for CP and PP

**Fig 1:**
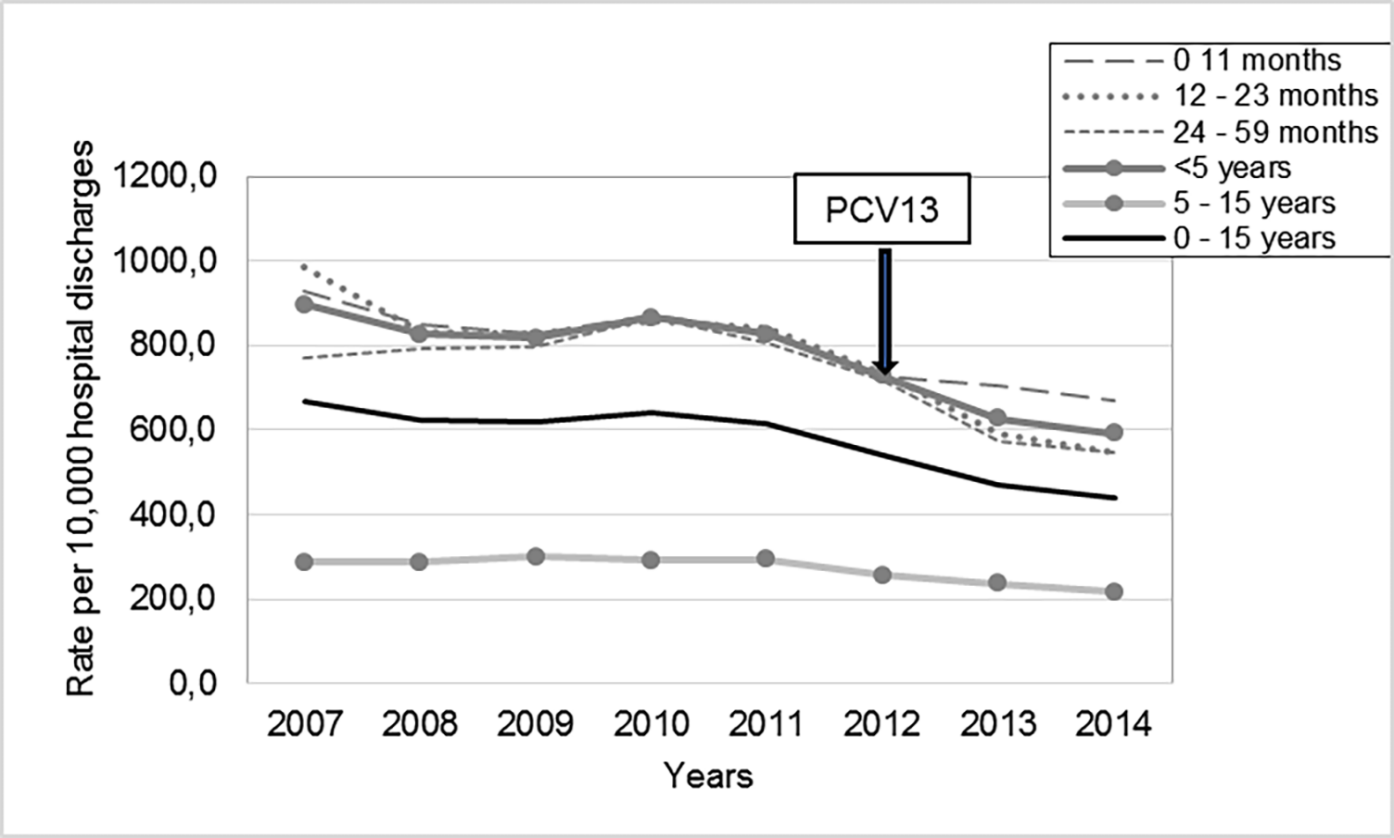
Discharge rate of consolidated pneumonia by age group during 2007–2014.

Analysis of discharge rates by age group showed statistically significant reduction in CP in children <5 years [% reduction (95%CI)]: 10.2% (6.3–14.0) in the intervention year (2012) and 24.8% (21.2–28.2) in the postVp, mainly in the age group from 12 to 23 months during both periods. In children >5 years, a 13.8% (5.2–21.6) reduction was observed in the postVp (Table 1 and Fig 1).

**Table 1:** Consolidated pneumonia discharge rates by age. PCV13 vaccination impact.

Age Group	Pre-Vaccination Period: 2007–2011 (Annual average)			Intervention Period: 2012				Post-Vaccination Period: 2013–14 (Annual Average)			
	Discharges	n	Discharge Rates^a^	Discharges	N	Discharge Rates^a^	% Reduction (95% CI)	Discharges	n	Discharge Rates^a^	% Reduction (95% CI)
0–11 months	23029	1873	813.32	22487	1638	728.42	10.4 (4.3; 16.2)	18271	1252	685.24	15.8 (9.5; 21.6)
12–23 months	13817	1309	947.38	13492	995	737.47	22.2 (15.5; 28.3)	14138	803	567.97	40.1 (34.5; 45.1)
24–59 months	18423	1298	704.55	17990	1294	719.29	2.1 (-5.8; 9.3)	15717	880	559.90	20.5 (13.4; 27.1)
<5 years	55269	4480	810.58	53969	3927	727.64	10.2 (6.3; 14.0)	48126	2935	609.86	24.8 (21.3; 28.2)
5–15 years	36846	971	263.53	35980	921	255.98	2.9 (-6.3; 11.2)	32926	748	227.18	13.8 (5.2; 21.6)
0–15 years	92115	5451	591.76	89949	4848	538.97	8.9 (5.3; 12.4)	81052	3683	454.40	23.2 (19.9; 26.4)

^a^Per 10.000 hospital discharges

**Fig 2:**
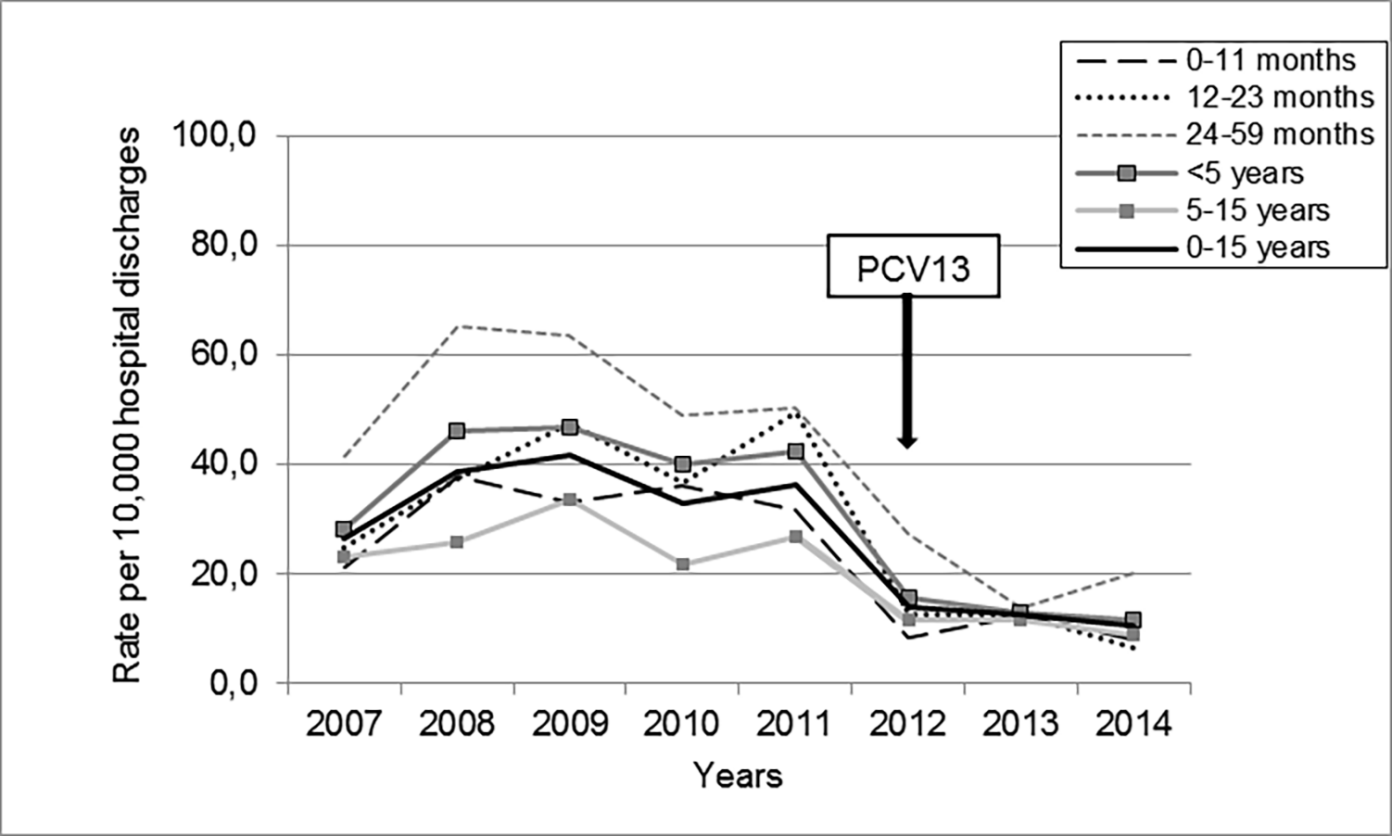
Discharge rate of pneumococcal pneumonia by age group during 2007–2014.

PP rate was significantly reduced for all age groups during both the intervention year and the postVp (Table 2 and Fig 2).

**Table 2:** Pneumococcal pneumonia discharge rates by age. PCV13 vaccination impact.

Age Group	Pre-Vaccination Period: 2007–2011 (Annual average)			Intervention Period: 2012				Post-Vaccination Period: 2013–14 (Annual Average)			
	Discharges	n	Discharge Rates^a^	Discharges	n	Discharge Rates^a^	% Reduction (95% CI)	Discharges	n	Discharge Rates^a^	% Reduction (95% CI)
0–11 months	23029	69	29.96	22487	19	8.45	71.8 (53.1; 83.0)	18271	18.5	10.13	66.2 (43.6; 79.8)
12–23 months	13817	59	42.70	13492	17	12.60	70.5 (40.4; 82.8)	14138	13.5	9.55	77.6 (59.6; 87.6)
24–59 months	18423	87	47.22	17990	49	27.24	42.3 (18.2; 59.4)	15717	26.5	16.86	64.3 (44.9; 76.9)
<5 years	55269	215	38.90	53969	85	15.75	59.5 (48.0; 68.5)	48126	58.5	12.16	68.8 (58.3; 76.6)
5–15 years	36846	87	23.61	35980	41	11.40	51.7 (30.0; 66.7)	32926	34	10.33	56.3 (35.0; 70.6)
0–15 years	92115	302	32.79	89949	126	14.01	52.3 (47.4; 65.3)	81052	92.5	11.41	65.0 (55.8; 72.3)

^a^Per 10.000 hospital discharges

### Epidemiological-clinical patterns of PP cases after PCV13 vaccine introduction

Epidemiological-clinical data of 297 patients with PP were obtained; 59.3% males, mean age 54.8 months (standard deviation 45.1), median age 45.0 months (range 0–188); 53.2% had underlying illnesses, of which chronic respiratory infections were the most frequent in 27.9%; PCV13 coverage rate in children <2 years of age with vaccination record was 42.9% in 2012 and 84.5% in the postVp.

Clinical characteristics of patients with PP, and penicillin susceptibility of pneumococcal isolates in the intervention year and in the postVp are described in Table 3. There were no significant differences in the patient characteristics during the 3-year follow-up.

**Table 3:** Pneumococcal pneumonia cases features after PCV13 vaccine introduction.

Features	Total		Intervention Period 2012		Post-Vaccination Period 2013–2014		p
	N	%	N	%	n	%	
Pneumococcal Pneumonia	297		106		191		
Penicillin resistant S.peumoniae	10	3.4	6	5.7	4	2.1	0.175
Intermediate Resistance	2	0.7	2	1.9	0	0	
Age <2 years	93	31.3	29	27.4	64	33.5	0.335
Underlying disease	158	53.2	59	55.7	99	51.8	0.609
Malnutrition	23	7.7	10	9.4	13	6.8	0.558
Recent acute respiratory disease	82	27.6	24	22.6	58	30.4	0.197
Previous antibiotics (last 3 months)	55	18.5	22	20.8	33	17.3	0.560
Previous hospitalizations (last year)	110	37.0	40	37.7	70	36.6	0.952
Complications	177	59.6	63	59.4	114	59.7	1.000
Pleural effusion/empyema	152	51.2	59	55.7	93	4.7	0.303
Necrotizing pneumonia	24	8.1	12	11.3	12	6.3	0.192
Pneumothorax	10	3.4	2	1.9	8	4.2	0.503
Atelectasis	9	3.0	3	2.8	6	3.1	1.000
Others	**9**	3.0	3	2.8	6	3.1	1.000
Case-fatality rate	10	3.4	2	1.9	8	4.2	0.503

Global case-fatality rate was 3.4% and was greater in children <2 years of age (6.5%; 6/93) than in children 2 to 15 years (2.0%; 4/204), although the difference was not statistically significant (p = 0.076). No significant difference was found between case-fatality rate during the intervention period and that of the postVp (p = 0.503).

### Microbiological diagnosis and serotype distribution

Microbiological diagnosis was performed in 318 samples from 297 patients: blood (237; 74.5%) and pleural fluid (81; 25.5%); in 21 patients, Sp was isolated in both blood and pleural fluid. Penicillin resistance (PR) was 3.4%, intermediate susceptibility was 0.7%.

**Fig 3:**
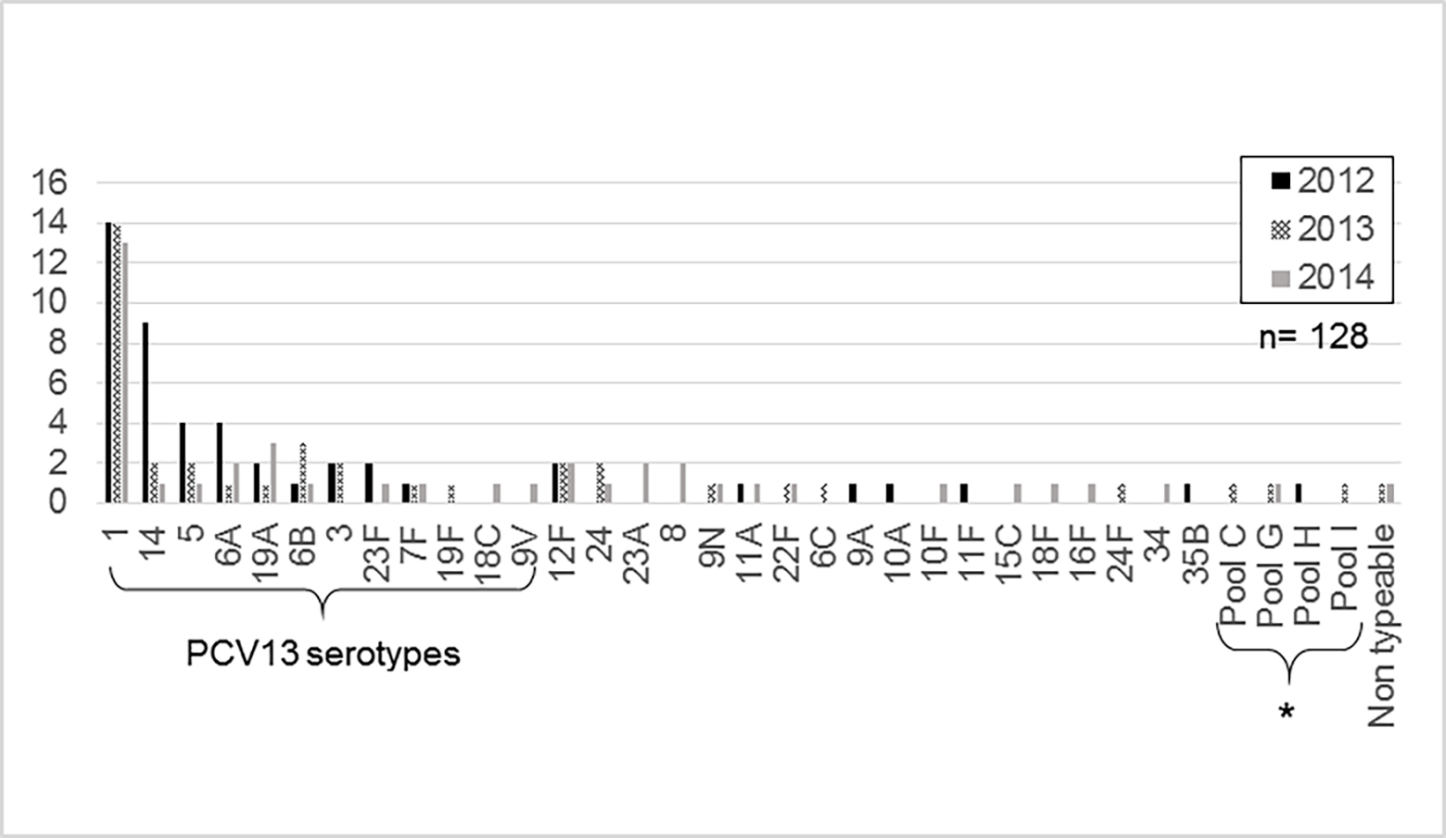
Pneumococcal pneumonia cases after PCV13 vaccine introduction: Distribution of *S*. *pneumoniae* serotypes. *Partially typeable pools: C [24, 31 ó 40], G [29,34,35,42 or 47], H [13 ó 28], I [38, 25F or 25A].

Sp isolates were serotyped in 43.1% of patients (128/297) Fig 3.

Serotypes included in PCV13 decreased from 83.0% (39/47) in 2012 to 64.2% (52/81) in the postVp, p = 0.039.

The most frequently identified serotype was 1 which did not show significant changes over the 3-year study period. Serotype 14 was the second most common serotype, decreasing significantly from 9 isolations in 2012, to 2 in 2013 and 1 in 2014 (9/47 to 3/81; p = 0.008).

## Discussion

Estimating the burden of CP in the pediatric population under WHO guidelines and specific rates of microbiologically confirmed PP are appropriate criteria to evaluate the impact of PCVs programs. In this study, as in other national and Latin American studies, a decrease in the burden of pneumonia in children was observed after the introduction of PCV13 in the national calendar [19–23].

An overall reduction in CP rates in children under 5 years was observed, more remarkably in the postVp; a greater impact was observed in young children 12 to 23 months of age during both periods in spite of a fall in booster dose coverage in the second year of life.

CP also decreased significantly in Infants < one year of age in both periods but less than in the group aged 12 to 23 months. This could be related to a greater incidence of respiratory viral infections in infants under 12 months and a direct relationship between bacterial etiology and age [24].

On the other hand, several studies have described that viral infections can cause localized alveolar infiltrate (lung consolidation) [25, 26], and other authors have reported increased incidence of pneumonia in association with viral diseases, such as influenza, parainfluenza, RSV, adenovirus, or metapneumovirus [27–32].

In a case control study, upper respiratory infections caused by viruses like influenza and parainfluenza correlated with acquisition of new serotypes of Sp in children [33].

In our study almost 30% of patients with PP had a history of a recent acute respiratory infection. Regarding the impact of PCV13 on PP rates, a significant reduction was observed among children in both vaccinated and nonvaccinated age-groups. Several studies describe this decline coinciding with emergence of less frequent non-vaccine serotypes, reinforcing the need for continued PP and invasive pneumococcal disease surveillance [34, 35].

As Weinberger et al. [36] suggest, the duration of the post-vaccination follow-up period can influence the magnitude of changes in disease incidence. There appears to be a lag between vaccine introduction and an increase in invasive pneumococcal disease (IPD) caused by non-vaccine serotypes. This could be attributed to the amount of time required to reach full vaccine coverage and for vaccine serotypes to be eliminated, especially through herd effects in the general population.

One weakness of our study is the lack of information of pneumococcal serotypes in the preVp as well as scarce epidemiological-clinical data in this period. Also, in only about half of the patients could a serotype be identified, mainly due to logistic difficulties in sending samples to the reference laboratory, or in other cases because PCR technique was used. However, a significant decrease in vaccine serotypes could be shown, as well as a relative increase in non-vaccine serotypes in the postVp.

It is important to highlight that PCV13 covers 85% of serotypes involved in all IPD and 88.4% of those causing pneumonia according to data 2011 from SIREVA II (Network Surveillance System for the Bacterial Agents Responsible for Pneumonia and Meningitis) [37].

Serotype 1, which is included in PCV13, was the most commonly isolated serotype during the 3-year study period, a phenomenon also described in other countries with other vaccine serotypes. During 2011, in a surveillance study for IPD at eight children’s hospitals in the United States, serotypes 19A and 7F remained the most common ones isolated from children hospitalized with pneumonia [38]. In another study, serotype 3 was isolated from pleural fluid specimens in 15 of 20 children with pneumococcal empyema, one-third of whom had received PCV13 [39]. In addition, serotype 1 is of special interest since it has exhibited a secular trend with long-term fluctuations, as has been reported in other countries [40]. Serotypes 19A, 7F and 3 remained low in prevalence, as historically seen in Argentina [41].

Regarding serotype 14, it is important to highlight low recovery of this serotype in the postVp, taking into account that it had been the predominant cause of IPD, mainly pneumonia, in the pre-vaccination era in our country [41, 42].

Sp resistance to multiple antibiotics is an important clinical problem. However, in our study resistance to penicillin was low. This could be related with changes in definitions in susceptibility cut-offs [18] as well as to the introduction of PCV13 to the program.

Sixty percent of patients with PP showed complications, a percentage slightly higher than levels reported in the literature, which range from 40 to 50% [43, 44], and included the classically reported complications [45–47], mainly pleural effusion/empyema, followed by necrotizing pneumonia, pneumothorax and atelectasis.

In the United States, PP complicated by pleural effusion/empyema increased in frequency in the period prior to the universal introduction of pneumococcal conjugate vaccine in 2000, and continued increasing after vaccine introduction [48, 49].

In our study, in the postVp, the decrease of PP cases was not associated with an increase in pleural effusion or other complications. Most of the children with PP, even those with complicated PP, recovered without sequelae.

PP case fatality rate (3.4%) was similar to that described in the literature, and was higher in children <2 years [50].

## Conclusions

After the introduction of PCV13 into the National Immunization Program, our study has shown a significant decrease in the burden of pneumonia in children, mainly in the group 12 to 23 months of age, a significant decrease in vaccine serotypes as well as a rise in non-vaccine serotypes, and low pneumococcal resistance to penicillin.

The decrease in PP cases was not associated with an increase in pleural effusion or other complications.

## Supporting information

S1 File PCV13 vaccination impact on pneumonia discharge rates. Pneumococcal Pneumonia cases features.
(DOCX)

## References

[1] Bryce J, Boschi-Pinto C, Shibuya K, Black RE, and the WHO Child Health Epidemiology Reference Group. WHO estimates of the causes of death in children. Lancet. 2005;365(9465):1147–52. 10.1016/S0140-6736(05)71877-8 15794969

[2] Rudan I, Boschi-Pinto C, Biloglav Z, Mulholland K, Campbell H. Epidemiology and etiology of childhood pneumonia. 2008;86(5):408–416. 10.2471/BLT.07.048769 18545744 PMC2647437

[3] Harris M, Clark J, Coote N, Fletcher P, Harnden A, McKean M, et al British Thoracic Society guidelines for the management of community acquired pneumonia in children: update 2011. Thorax. 2011; 66 (Suppl. 2):ii1–23. 21903691 10.1136/thoraxjnl-2011-200598

[4] World Health Organization. Pneumococcal vaccines WHO position paper—2012.Wkly Epidemiol Rec. 2012;87:129–144. 24340399

[5] Rudan I, O’Brien KL, Nair H, Liu L, Theodoratou E, Qazi S, et al Epidemiology and etiology of childhood pneumonia in 2010: estimates of incidence, severe morbidity, mortality, underlying risk factors and causative pathogens for 192 countries. Journal of Global Health. 2013;3(1):010401 10.7189/jogh.03.010401 23826505 PMC3700032

[6] Tregnaghi M, Cevallos A, Ruttimann R, Ussher J, Tregnaghi P, Peeters P, et al Active epidemiologic surveillance of pneumonia and invasive pneumococcal disease in ambulatory and hospitalized infants in Cordoba, Argentina. Pediatr Infect Dis J. 2006;25 (4):370–2. 10.1097/01.inf.0000208368.67448.51 16567994

[7] Hortal M, Estevan M, Iraola I, De Mucio B. A population-based assessment of the disease burden of consolidated pneumonia in hospitalized children under five years of age. Int. J Infect Dis. 2007;11(3):273–7. 10.1016/j.ijid.2006.05.006 16997592

[8] Lagos R, Muñoz A, San Martin O, Maldonado A, Hormazabal JC, Blackwelder WC, et al Age- and serotype-specific pediatric invasive pneumococcal disease: insights from systematic surveillance in Santiago, Chile, 1994–2007. J Infect Dis. 2008;198(12):1809–1817. 10.1086/593334 18959497

[9] Ruvinsky R, Regueira M, Fossati MS, Gagetti P, Pace J, Rodriguez M, et al Surveillance of invasive Streptococcus pneumoniae in Argentina 1994–2007: Changes in serotype distribution, serotype coverage of pneumococcus conjugate vaccines and antibiotic resistance. J Pediatr Infect Dis. 2010;5(3):263–9.

[10] Black S, Shinefield H, Fireman B, Lewis E, Ray P, Hansen JR, et al Efficacy, safety and immunogenicity of heptavalent pneumococcal conjugate vaccine in children. Northern California Kaiser Permanente Vaccine Study Center Group. Pediatr Inf Dis J. 2000;19(3):187–195. 10.1097/00006454-200003000-00003 10749457

[11] Klugman KP, Madhi SA, Huebner RE, Kohberger R, Mbelle N, Pierce N, Vaccine Trialists Group. A Trial of a 9-Valent Pneumococcal Conjugate Vaccine in Children with and those without HIV Infection. N Engl J Med. 2003;349(14):1341–48. 10.1056/NEJMoa035060 14523142

[12] Cutts FT, Saman SM, Enwere G, Jaffar S, Levine OS, Okoko JB, et al Efficacy of nine-valent pneumococcal conjugate vaccine against pneumonia and invasive pneumococcal disease in The Gambia: randomized, double-blind, placebo-controlled Trial. Lancet. 2005;365(9465):1139–46. 10.1016/S0140-6736(05)71876-6 15794968

[13] Lucero MG, Nohynek H, Williams G, Tallo V, Simões EA, Lupisan S, et al Efficacy of an 11-Valent Pneumococcal Conjugate Vaccine Against Radiologically Confirmed Pneumonia Among Children Less Than 2 Years of Age in the Philippines. A Randomized, Double-Blind, Placebo-Controlled Trial. Pediatr Inf Dis J. 2009;28(6):455–462. 10.1097/INF.0b013e31819637af 19483514

[14] McElligott M, Vickers I, Cafferkey M, Cunney R, Humphreys H. Non-invasive pneumococcal serotypes and antimicrobial susceptibilities in a paediatric hospital in the era of conjugate vaccines. Vaccine. 2014;32(28):3495–3500. 10.1016/j.vaccine.2014.04.047 24795223

[15] Urueña A, Pippo T, Betelu MS, Virgilio F, Giglio N, Gentile A, et al Cost-effectiveness analysis of the 10- and 13-valent pneumococcal conjugate vaccines in Argentina. Vaccine. 2011;29(31):4963–4972. 10.1016/j.vaccine.2011.04.111 21621575

[16] WHO. WHO vaccine-preventable diseases: monitoring system. 2016 global summary Coverage time series for Argentina. Available at: http://apps.who.int/immunization_monitoring/globalsummary/coverages?c=ARG. Accessed April 6, 2017.

[17] McIntosh K. Community-acquired pneumonia in children. N Engl J Med. 2002; 346: 429–37. 10.1056/NEJMra011994 11832532

[18] MIC Interpretive Standards for Streptococcus pneumoniae. Clinical Laboratory Standards Institute (CLSI). 2008;28:123.

[19] Gentile A, Juárez MV, Lución MF, Romanin V, Giglio N, Bakir J. Influence of Respiratory Viruses on the Evaluation of 13-Valent Pneumococcal Conjugate Vaccine Effectiveness in Children Under 5 Years Old: A Time-Series Study 2001–2014. Arch argent pediatr. 2015;113(4):310–16. 10.5546/aap.2015.310 26172005

[20] Gentile A, Bakir J, Bialorus L, Caruso L, Mirra D, Santander C, et al Impacto de la vacuna neumocócica conjugada 13-valente en la incidencia de neumonía consolidante en menores de 5 años en el partido de Pilar, Buenos Aires: estudio de base poblacional. Arch argent pediatr. 2015;113(6):502–9. 10.5546/aap.2015.502 26593795

[21] Hortal M, Estevan M, Laurani H, Iraola I, Meny M, Paysandú-Salto Study Group. Hospitalized children with pneumonia in Uruguay: pre and post introduction of 7 and 13-valent pneumococcal conjugated vaccines into the National Immunization Program. Vaccine. 2012; 30(33): 4934–38. 10.1016/j.vaccine.2012.05.054 22664222

[22] Afonso ET, Minamisava R, Bierrenbach AL, Escalante JJ, Alencar AP, Domingues CM, et al Effect of 10-valent pneumococcal vaccine on pneumonia among children, Brazil. Emerg Infect Dis. 2013;19(4):589–97 10.3201/eid1904.121198 23628462 PMC3647414

[23] Pírez MC, Algorta G, Cedrés A, Sobrero H, Varela A, Giachetto G, et al Impact of universal pneumococcal vaccination on hospitalizations for pneumonia and meningitis in children in Montevideo, Uruguay. Pediatr Infect Dis J. 2011; 30(8):669–74. 10.1097/INF.0b013e3182152bf1 21407145

[24] Comité de infecciones respiratorias de la Sociedad Latinoamericana de Infectología Pediátrica. Consenso de la Sociedad Latinoamericana de Infectología Pediátrica sobre neumonía adquirida en la comunidad. Rev Enferm Infecc Pediatr. 2010;24(94):1–23.

[25] Lehtinen P, Jartti T, Virkki R, Vuorinen T, Leinonen M, Peltola V, et al Bacterial coinfections in children with viral wheezing. Eur J Clin Microbiol Infect Dis. 2006;25(7):463–9. 10.1007/s10096-006-0166-3 16819619 PMC7088417

[26] Ruuskanen O, Lahti E, Jennings LC, Murdoch DR. Viral pneumonia. Lancet. 2011;377(9773):1264–75. 10.1016/S0140-6736(10)61459-6 21435708 PMC7138033

[27] Gentile A, Bakir J, Russ C, Ruvinsky S, Ensinck G, Falaschi A, et al Estudio de las enfermedades respiratorias por virus Influenza A H1N1 (pH1N1) en niños internados durante el año de la pandemia. Experiencia de 34 centros en la Argentina. Arch argent pediatr. 2011; 109(3):198–203. 10.1590/S0325-00752011000300003 21660384

[28] Gentile A, Giglio N, Romanin VS, Juarez MV, Lucion MF, Bakir J. Influence of respiratory viruses on the evaluation of the 13-valent pneumococcal conjugate vaccine effectiveness in children under 5 years old: A time-series study for the 2001–2013 period. Arch argent pediatr. 2015; 113(4): 310–6. 10.5546/aap.2015.310 26172005

[29] Madhi SA, Klugman KP, Vaccine Trialist Group. A role for Streptococcus pneumoniae in virus-associated pneumonia. Nat Med. 2004; 10(8):811–3. 10.1038/nm1077 15247911 PMC7095883

[30] Madhi SA, Ludewick H, Kuwanda L, van Niekerk N, Cutland C, Little T, et al Pneumococcal coinfection with human metapneumovirus. J Infect Dis. 2006; 193(9):1236–43. 10.1086/503053 16586360

[31] Ampofo K, Bender J, Sheng X, Korgenski K, Daly J, Pavia AT, et al Seasonal invasive pneumococcal disease in children: role of preceding respiratory viral infection. Pediatrics. 2008; 122(2):229–37. 10.1542/peds.2007-3192 18676537

[32] O'Brien KL, Walters MI, Sellman J, Quinlisk P, Regnery H, Schwartz B, et al Severe pneumococcal pneumonia in previously healthy children: the role of preceding influenza infection. Clin Infect Dis. 2000; 30(5):784–9. 10.1086/313772 10816149

[33] Grijalva CG, Griffin MR, Edwards KM, Williams JV, Gil AI, Verastegui H, et al The role of influenza and parainfluenza infections in nasopharyngeal pneumococcal acquisition among young children. Clin Infect Dis. 2014; 58(10):1369–1376. 10.1093/cid/ciu148 24621951 PMC4001292

[34] Galanis I, Lindstrand A, Darenberg J, Browall S, Nannapaneni P, Sjöström K, et al Effects of PCV7 and PCV13 on invasive pneumococcal disease and carriage in Stockholm, Sweden. Eur Respir J. 2016;47(4):1208–18. 10.1183/13993003.01451-2015 26797033 PMC4819883

[35] Moore MR, Link-Gelles R, Schaffner W, Lynfield R, Lexau C, Bennett NM, et al Effect of use of 13-valent pneumococcal conjugate vaccine in children on invasive pneumococcal disease in children and adults in the USA: analysis of multisite, population-based surveillance. Lancet Infect Dis. 2015;15(3):301–9. 10.1016/S1473-3099(14)71081-3 25656600 PMC4876855

[36] Weinberger DM, Malley R, Lipsitch M. Serotype replacement in disease following pneumococcal vaccination: A discussion of the evidence. Lancet. 2011;378(9807):1962–1973. 10.1016/S0140-6736(10)62225-8 21492929 PMC3256741

[37] Organización Panamericana de la Salud. Informe Regional de SIREVA II, 2012: datos por país y por grupos de edad sobre las características de los aislamientos de Streptococcus pneumoniae, Haemophilus influenzae y Neisseria meningitidis, en procesos invasores. 2013; Washington DC. Available at: http://www.paho.org/hq/index.php?option=com_docman&task=doc_download&gid=22372&Itemid=270〈=es. Accessed April 30, 2017.

[38] Kaplan SL, Barson WJ, Lin PL, Romero JR, Bradley JS, Tan TQ, et al Early trends for invasive pneumococcal infections in children after the introduction of the 13-valent pneumococcal conjugate vaccine. Pediatr Infect Dis J. 2013; 32(3):203–7. 10.1097/INF.0b013e318275614b 23558320

[39] Antachopoulos C, Tsolia MN, Tzanakaki G, Xirogianni A, Dedousi O, Markou G, et al Parapneumonic pleural effusions caused by Streptococcus pneumoniae serotype 3 in children immunized with 13-valent conjugated pneumococcal vaccine. Pediatr Infect Dis J. 2014; 33(1):81–3. 10.1097/INF.0000000000000041 24172850

[40] Fenoll A, Granizo JJ, Aguilar L, Gimenez MJ, Aragoneses-Fenoll L, Hanquet G, et al Temporal Trends of Invasive Streptococcus pneumoniae Serotypes and Antimicrobial Resistance Patterns in Spain from 1979 to 2007. J Clin Microbiol. 2009;47(4):1012–20. 10.1128/JCM.01454-08 19225097 PMC2668361

[41] Ruvinsky R, Gentile A, Regueira M, Corso A, Pace J, Bakir J et al Infecciones invasivas por Streptococcus pneumoniae: Estudio epidemiológico e importancia del desarrollo de un sistema de vigilancia. Rev chil pediatr. 2004;75(1):77–9.

[42] Mayoral C, Baroni MR, Giani R, Virgolini S, Zurbriggen L, Regueira M. Distribución de serotipos de Streptococcus pneumoniae aislados de infecciones invasoras en el Hospital de Niños de Santa Fe. Revista argentina de microbiología. 2008;40(1):13–6. 18669047

[43] Tan TQ, Mason EO Jr, Wald ER, Barson WJ, Schutze GE, Bradley JS, et al Clinical characteristics of children with complicated pneumonia caused by Streptococcus pneumoniae. Pediatrics. 2002;110(1):1–6. 12093940 10.1542/peds.110.1.1

[44] Wexler ID, Knoll S, Picard E, Villa Y, Shoseyov D, Engelhard D, et al Clinical characteristics and outcome of complicated pneumococcal pneumonia in a pediatric population. Pediatr Pulmonol. 2006; 41(8):726–34. 10.1002/ppul.20383 16779839

[45] Byington CL, Spencer LY, Johnson TA, Pavia AT, Allen D, Mason EO, et al An epidemiological investigation of a sustained high rate of pediatric parapneumonic empyema: risk factors and microbiological associations. Clin Infect Dis. 2002; 34(4):434–40. 10.1086/338460 11797168

[46] Ramphul N, Eastham KM, Freeman R, Eltringham G, Kearns AM, Leeming JP, et al Cavitatory lung disease complicating empyema in children. Pediatr Pulmonol. 2006; 41(8):750–3. 10.1002/ppul.20434 16779851

[47] Bender JM, Ampofo K, Korgenski K, Daly J, Pavia AT, Mason EO, et al Pneumococcal necrotizing pneumonia in Utah: does serotype matter? Clin Infect Dis. 2008; 46(9):1346–52. 10.1086/586747 18419434 PMC3673544

[48] Byington CL, Korgenski K, Daly J, Ampofo K, Pavia A, Mason EO. Impact of the pneumococcal conjugate vaccine on pneumococcal parapneumonic empyema.Pediatr Infect Dis J. 2006; 25(3):250–4. 10.1097/01.inf.0000202137.37642.ab 16511389

[49] Grijalva CG, Nuorti JP, Zhu Y, Griffin MR. Increasing incidence of empyema complicating childhood community-acquired pneumonia in the United States. Clin Infect Dis. 2010; 50(6):805–13. 10.1086/650573 20166818 PMC4696869

[50] Feikin DR, Schuchat A, Kolczak M, Barrett NL, Harrison LH, Lefkowitz L, et al Mortality from invasive pneumococcal pneumonia in the era of antibiotic resistance, 1995–1997. Am J Public Health. 2000; 90(2):223–9. 10667183 10.2105/ajph.90.2.223 PMC1446155

