# Supplementary material for: PCV13 vaccination impact: A multicenter study of pneumonia in 10 pediatric hospitals in Argentina
Source: PLoS One. 2018 Jul 18;13(7):e0199989. doi: 10.1371/journal.pone.0199989 (PMC6051625; doi:10.1371/journal.pone.0199989)
Supplement: S1 File — Pneumococcal Pneumonia cases features. (DOCX) [file pone.0199989.s001.docx]

**Table 1. Consolidated Pneumonia Discharge Rates by Age. PCV13 Vaccination Impact.**

| **Age Group** | **Pre-Vaccination Period: 2007 - 2011**  **(Annual average)** | | | **Intervention Period: 2012** | | | | **Post-Vaccination Period: 2013-14**  **(Annual Average)** | | | |
| --- | --- | --- | --- | --- | --- | --- | --- | --- | --- | --- | --- |
|  | **Discharges** | **n** | **Discharge Rates^a^** | **Discharges** | **N** | **Discharge Rates^a^** | **% Reduction**  **(95% CI)** | **Discharges** | **n** | **Discharge Rates^a^** | **Reduction**  **(95% CI)** |
| 0-11 months | 23029 | 1873 | 813.32 | 22487 | 1638 | 728.42 | 10.4 (4.3; 16.2) | 18271 | 1252 | 685.24 | 15.8 (9.5; 21.6) |
| 12-23 months | 13817 | 1309 | 947.38 | 13492 | 995 | 737.47 | 22.2 (15.5; 28.3) | 14138 | 803 | 567.97 | 40.1 (34.5; 45.1) |
| 24-59 months | 18423 | 1298 | 704.55 | 17990 | 1294 | 719.29 | 2.05 (-5.79; 9.31) | 15717 | 880 | 559.90 | 20,5 (13.4; 27.1) |
| <5 years | 55269 | 4480 | 810.58 | 53969 | 3927 | 727.64 | 10.2 (6.3; 14.0) | 48126 | 2935 | 609.86 | 24.8 (21.3; 28.2) |
| 5-15 years | 36846 | 971 | 263.53 | 35980 | 921 | 255.98 | 2.87 (-6.30; 11.24) | 32926 | 748 | 227.18 | 13.8 (5.2; 21.6) |
| 0-15 years | 92115 | 5451 | 591.76 | 89949 | 4848 | 538.97 | 8.9 (5.3; 12.4) | 81052 | 3683 | 454.40 | 23.2 (19.9; 26.4) |
| ^a^Per 10.000 hospital discharges  **Table 2. Pneumococcal Pneumonia Discharge Rates by Age. PCV13 Vaccination Impact.**   \| **Age Group** \| **Pre-Vaccination Period: 2007 - 2011**  **(Annual average)** \| \| \| **Intervention Period: 2012** \| \| \| \| **Post-Vaccination Period: 2013-14**  **(Annual Average)** \| \| \| \| \| --- \| --- \| --- \| --- \| --- \| --- \| --- \| --- \| --- \| --- \| --- \| --- \| \|  \| **Discharges** \| **n** \| **Discharge Rates^a^** \| **Discharges** \| **n** \| **Discharge Rates^a^** \| **% Reduction**  **(95% CI)** \| **Discharges** \| **n** \| **Discharge Rates^a^** \| **% Reduction**  **(95% CI)** \| \| 0-11 months \| 23029 \| 69 \| 29.96 \| 22487 \| 19 \| 8.45 \| 71.8 (53.1; 83.0) \| 18271 \| 18.5 \| 10.13 \| 66.2 (43.6; 79.8) \| \| 12-23 months \| 13817 \| 59 \| 42.70 \| 13492 \| 17 \| 12.60 \| 70.5 (40.4; 82.8) \| 14138 \| 13.5 \| 9.55 \| 77.6 (59.6; 87.6) \| \| 24-59 months \| 18423 \| 87 \| 47.22 \| 17990 \| 49 \| 27.24 \| 42.3 (18.2; 59.4) \| 15717 \| 26.5 \| 16.86 \| 64.3 (44.9; 76.9) \| \| <5 years \| 55269 \| 215 \| 38.90 \| 53969 \| 85 \| 15.75 \| 59.5 (48.0; 68.5) \| 48126 \| 58.5 \| 12.16 \| 68.8 (58.3; 76.6) \| \| 5-15 years \| 36846 \| 87 \| 23.61 \| 35980 \| 41 \| 11.40 \| 51.7 (30.0; 66.7) \| 32926 \| 34 \| 10.33 \| 56.3 (35.0; 70.6) \| \| 0-15 years \| 92115 \| 302 \| 32.79 \| 89949 \| 126 \| 14.01 \| 52.3 (47.4;65.3) \| 81052 \| 92.5 \| 11.41 \| 65.0 (55.8;72.3) \|   ^a^Per 10.000 hospital discharges  **Table 3. Pneumococcal Pneumonia cases features after PCV13 vaccine introduction.**   \| **Features** \| **Total** \| \| Intervention Period  2012 \| \| Post-Vaccination Period  2013-2014 \| \| p \| \| --- \| --- \| --- \| --- \| --- \| --- \| --- \| --- \| \| n \| % \| n \| % \| n \| % \| \| Pneumococcal Pneumonia \| 297 \|  \| 106 \|  \| 191 \|  \|  \| \| Penicillin resistant S.peumoniae \| 10 \| 3.4 \| 6 \| 5.7 \| 4 \| 2.1 \| 0.1752 \| \| Intermediate Resistence \| 2 \| 0.7 \| 2 \| 1.9 \| 0 \| 0 \|  \| \| Age <2 years \| 93 \| 31.3 \| 29 \| 27.4 \| 64 \| 33.5 \| 0.3349 \| \| Underlying disease \| 158 \| 53.2 \| 59 \| 55.7 \| 99 \| 51.8 \| 0.6086 \| \| Malnourishment \| 23 \| 7.7 \| 10 \| 9.4 \| 13 \| 6.8 \| 0.5584 \| \| Recent acute respiratory disease \| 82 \| 27.6 \| 24 \| 22.6 \| 58 \| 30.4 \| 0.1966 \| \| Previous antibiotics (last 3 months) \| 55 \| 18.5 \| 22 \| 20.8 \| 33 \| 17.3 \| 0.5598 \| \| Previous hospitalizations (last year) \| 110 \| 37.0 \| 40 \| 37.7 \| 70 \| 36.6 \| 0.9518 \| \| Complications \| 177 \| 59.6 \| 63 \| 59.4 \| 114 \| 59.7 \| 1.0000 \| \| Pleural effusion/empyema \| 152 \| 51.2 \| 59 \| 55.7 \| 93 \| 4.7 \| 0.3030 \| \| Necrotizing pneumonia \| 24 \| 8.1 \| 12 \| 11.3 \| 12 \| 6.3 \| 0.1922 \| \| Pneumothorax \| 10 \| 3.4 \| 2 \| 1.9 \| 8 \| 4.2 \| 0.5034 \| \| Atelectasis \| 9 \| 3.0 \| 3 \| 2.8 \| 6 \| 3.1 \| 1.0000 \| \| Others \| **9** \| 3.0 \| 3 \| 2.8 \| 6 \| 3.1 \| 1.0000 \| \| Case-fatality rate \| 10 \| 3.4 \| 2 \| 1.9 \| 8 \| 4.2 \| 0.5034 \| | | | | | | | | | | | |
